# Critical psychiatry: an embarrassing hangover from the 1970s?

**DOI:** 10.1192/bjb.2020.5

**Published:** 2020-12

**Authors:** Duncan B. Double

**Affiliations:** Trinity College Cambridge, University of Cambridge, UK

**Keywords:** Aetiology, philosophy, history of psychiatry, critical psychiatry, anti-psychiatry

## Abstract

Critical psychiatry is associated with anti-psychiatry and may therefore seem to be an embarrassing hangover from the 1970s. However, its essential position that functional mental illness should not be reduced to brain disease overlaps with historical debates in psychiatry more than is commonly appreciated. Three examples of non-reductive approaches, like critical psychiatry, in the history of psychiatry are considered.

The Critical Psychiatry Network has promoted critical psychiatry for the past 20 years.^1^ It is a small group of psychiatrists and although psychiatrists in general may be aware of its critique, it remains a minority perspective. In this sense, critical psychiatry still seems marginal to mainstream practice. This may be because of its association with so-called anti-psychiatry. Although the first modern use of ‘anti-psychiatry’ was probably by David Cooper in his book *Psychiatry and Anti-Psychiatry*,^2^ the term has generally been applied by mainstream psychiatry to denote criticism that it does not accept. For example, Martin Roth, when he was the first president of the Royal College of Psychiatrists, identified an international movement against psychiatry that he regarded as ‘anti-medical, anti-therapeutic, anti-institutional and anti-scientific’.^3^

## Psychiatry's response to anti-psychiatry

Anti-psychiatry, perhaps most associated with the names of Thomas Szasz and Ronald Laing, now tends to be seen as a passing phase in the history of psychiatry.^4^ In this sense it was an aberration, a discontinuity with the proper course of psychiatry. However, it is difficult to accept that there was no value in the approach and what may be more beneficial is to look for the continuities, rather than discontinuities, with orthodox psychiatry. In fact, anti-psychiatry is not a single perspective; for example, Szasz equally rejected both mainstream psychiatry and Laing's views.^5^

Part of how psychiatry was able to move on from anti-psychiatry was by trying to make psychiatric diagnoses more reliable; for example, through the American Psychiatric Association's publication of the DSM-III. Another way was by avoiding ideological disputes engendered by anti-psychiatry, by encouraging eclecticism in practice that includes psychosocial as well as biological factors.^6^ The challenge to a narrowly biomedical model, which was of such concern for anti-psychiatry, was thereby averted.

Mainstream psychiatry now tends to regard anti-psychiatry as a ‘straw man’ argument. It was sustained by the counter-culture of the 1960s and without this support it has waned away. Social cultural critiques, with which anti-psychiatry was aligned, are no longer academically respectable. Critical psychiatry, by apparently resurrecting anti-psychiatry ideas, may therefore seem like an embarrassing hangover from the 1970s.

## Historical foundations of critical psychiatry

The history of psychiatry can be understood as a conflict between two factions of somatic and psychic approaches rather than a simple chronological development. Critical psychiatry is part of this debate. The essential position of critical psychiatry is that functional mental illness should not be reduced to brain disease. Its challenge to reductionism and positivism, including mechanistic psychological approaches, creates a framework that focuses on the person and has ethical, therapeutic and political implications for clinical practice. This editorial focuses on conceptual issues, as the more practical aspects of critical psychiatry could be said to follow from its theoretical position. This is not meant to detract from these central practical concerns of critical psychiatry, such as the political and socioeconomic origins of mental illness.

The argument of critical psychiatry is that psychiatry can be practiced without taking the step of faith of believing that functional mental illness is owing to brain pathology. This anti-reductionism is primarily explanatory rather than ontological and does not undermine the importance of organic factors. Functional psychiatry could be said to be doomed to a kind of descriptivism rather than being able to become a mechanical natural science. Historically critical psychiatry may actually be more integrated with mainstream psychiatry than is commonly appreciated.

Modern psychiatry has its origins in the Enlightenment of the 18th century.^7^ The problem of knowledge began to shift away from philosophy toward science. Critical engagement of reason with itself created a descriptive approach to madness. Psychiatrists were originally called alienists, identifying mental alienation.

Descartes had separated the soul from the body, and, reacting against this Cartesianism, anthropology established itself as an independent discipline, concerned with the study of man as a psychophysical unity. In this context, medical psychology had its origin with two major variants of anthropological thinking.^8^ A medically orientated anthropology represented by Ernst Platner, among others, was one version, and can be seen as the root of biomedical psychiatry. Physiological knowledge of humans seemed to create the possibility of a natural scientific psychology. The other version was Immanuel Kant's pragmatic anthropology, which may be seen as the origin of critical psychiatry. Kant believed that a natural science psychology was impossible to realise in practice. Applying a physico-chemical mechanistic approach to life cannot accommodate the purposiveness of living beings. Organisms, unlike machines, are self-organising and self-reproducing systems. Kant was clear that it is futile to expect to be able to understand and explain life in terms of merely mechanical principles of nature.

Building on this proto-psychiatry, the first half of the 19th century saw the development of anatomoclinical understanding. Relating symptoms and signs to their underlying physical pathology was a major advance for medicine and still underlies our modern understanding of disease. Pathology emerged as a distinct discipline with autopsy findings of lesions in organs and tissues being related to clinical examination at the bedside. Histological studies established cellular abnormalities for disease.

Applying this anatomoclinical method to psychiatry was not as successful because it was not always easy to relate mental conditions to underlying brain pathology. The enthusiastic search for anatomical localisation in psychiatry led to fanciful notions later in the 19th century. For example, Theodor Meynert (1833–1892) delineated various ‘fibre-systems’ in the brain and deduced functions for these ‘pathways’. Meynert's research may have appeared so successful because it seemed to give a material explanation of the basis of mental illness. Despite his skills in brain dissection, his theories were not based on empirical findings. They were eventually attacked and labelled as ‘brain mythology’, particularly after his death.

It was eventually established that dementia paralytica was a late consequence of syphilis. Senile dementia was also seen as having a physical cause such as Alzheimer's disease. Focal abnormalities in the brain were identified and physical causes of learning disability were recognised. However, most psychopathology is functional, in the sense that there are no structural abnormalities in the brain.

Wilhelm Griesinger (1817–1868) was dedicated to the idea of the pathology and therapy of mental diseases as a mechanical natural science, although he remained aware of the gap between this ideal and reality. Nonetheless he set the trend for this positivist biomedical understanding that has dominated psychiatry since the middle of the 19th century. His aphorism that ‘mental diseases are brain diseases’ could be seen as the origin of modern biomedical psychiatry with its wish to find a physicalist basis for mental illness. Such a positivist reduction of mental illness to brain disease is what causes such concern for critical psychiatry.

This historical narrative is necessarily selective and schematic. It is more of a genealogy, attempting to make the origins of critical psychiatry intelligible. Psychiatry and its critical version had their origins at the same time in medical psychology. Incorporating the anatomoclinical way of understanding disease into psychiatry, particularly following Griesinger, has eclipsed a more critical understanding of mental illness. There have, nonetheless, been non-reductive approaches in modern psychiatry that amount to a critical position. For reasons of space, this editorial will consider just three examples: Ernst von Feuchtersleben, Adolf Meyer and George Engel.

## The forgotten psychiatrist Ernst von Feuchtersleben

In the same year, 1845, that saw the publication in German of the book that gave Wilhelm Griesinger his reputation in psychiatry, Ernst von Feuchtersleben produced his psychiatric textbook^9^ based on his lectures. Following Kant, he recognised that the mind–brain problem is an enigma, which can never be solved. He was aware of the somatic bias in medicine and one of the aims of his lectures was to encourage young physicians to study its psychical element. As far as he was concerned, all physicians should have a clear understanding of the relationship between mind and body.

Feuchtersleben took a holistic approach to medical psychology. Materialism, in the sense of reducing mind to body, as far as he was concerned, explains nothing because such reductionism leads to the loss of meaning of human action. Mental illness is deduced rather from the relationship of mind and body without necessarily being able to explain this relationship. There is a limit to the natural scientific understanding of mental life.

Philipp Carl Hartmann, his teacher and Chair of General Pathology, Therapy and Materia Medica at the Vienna Medical School, influenced Feuchtersleben.^10^ Hartmann's understanding of disease as a dynamic process was a corrective to the physicalist perspective. Although both Hartmann and Feuchtersleben of course recognised that mental activity has a physical basis, they were clear that physiology is not able to derive the activities of the mind completely from the laws of the physical world. Despite the success of Feuchtersleben's book, biomedical approaches became more dominant and his psychosomatic viewpoint had no impact in the second half of the 19th century.

## Adolf Meyer's psychobiology

Adolf Meyer was regarded as the Dean of American psychiatry in the first half of the 20th century. His approach, called psychobiology,^11^ has an integrated understanding of mind and brain. Meyer began his career as a pathologist and moved into the clinical field, standardising procedures for history-taking and mental state examination. Psychopathology needs to be studied functionally in experiences and social interactions rather than organically at the level of neurobiology. Psychobiology was not an aetiological psychiatry, in the sense of providing psychoanalytical mechanisms or Kraepelinian disease entities.

Meyer viewed mental activity and brain activity as a single biological response. Mental dysfunction, as much as brain disease, is a medical condition resulting from pathological processes. As far as Meyer was concerned, functional mental illnesses are failed adaptations, rather than distinct brain diseases. He was fond of calling a ‘neurologizing tautology’ any attempt to reduce mental illness to brain disease.

Meyer's ideas never really take hold as a systematic theory of psychiatry. This was partly because of his pragmatic compromising attitude. He was prepared to accommodate all perspectives in psychiatry even if he disagreed with them. He recognised this himself in a heartfelt note he wrote a few years before he died, saying, ‘I should have made myself clear and in outspoken *opposition*, instead of a mild semblance of harmony’.^12^

## George Engel's biopsychosocial model

George Engel's biopsychosocial model^13^ to integrate biological, psychological and social factors in medicine and psychiatry was a deliberate challenge to biomedical reductionism. Engel acknowledged the historical significance for his integrated and holistic model of the work of Adolf Meyer. He recognised the difficulties in overcoming the power of the prevailing biomedical structure, whose dogmatism he thought needed to be neutralised. As far as he was concerned, doctors had become insensitive to the personal problems of patients and were preoccupied with procedures. This was a crisis for the whole of medicine, not just psychiatry. An integrated understanding of the whole person, including emotional needs and life issues, forms the basis for patient-centred medicine.

The biopsychosocial model accepts the inherent uncertainty in psychiatric and medical practice. By contrast, the biomedical perspective seems to have an advantage because of its perceived potential for certainty in the understanding of mental disorder. The biopsychosocial model can be seen as too vague by comparison.

Further, the biopsychosocial model is often used in an eclectic way in current psychiatric practice. It is commonly said that biological, psychological and social must all be taken into account in psychiatric assessment, as though all three are more or less equally relevant in all cases and at all times. This ill-defined basis for practice may create theoretical inconsistency, such as viewing more minor psychological disorder as psychosocial, whereas more severe mental illness is identified as biological in origin. It may also lead to the combination of psychotherapy and biological treatments without any systematic theory to support such a strategy. This eclecticism has been critiqued^14^ and does seem to have outlived its usefulness.

In fact, Engel's original version of the biopsychosocial model was not eclectic and eclecticism has more to do with the mainstream response to anti-psychiatry.^15^ The conflict created by the split between biomedical and biopsychosocial models has encouraged the compromise of eclecticism to avoid ideological argument.

## Conclusion

To be clear, critical psychiatry is encouraging the integration of mind and body, not their separation. The brain is the origin of the mind and minds are enabled but not reducible to brains. In other words, mental disorders show *through* the brain but not necessarily *in* the brain. Critical psychiatry argues that believing that functional mental illness is a brain disease is more like a faith that doctors are obliged to believe rather than a scientific position.

As demonstrated with three examples, this essential position of critical psychiatry has been expressed in the history of modern psychiatry. At the same time as Griesinger was steering psychiatry toward a positivist understanding of mental illness, Feuchtersleben based psychiatry on Kant's critical philosophy. Meyer's psychobiology provides a legitimate theoretical framework for critical psychiatry, although any neo-Meyerian position must take into account Meyer's tendency to compromise and cannot simply be a restatement of his legacy. Engel's biopsychosocial model also provides a valid anti-reductionist position for critical psychiatry, although it should not be associated with the eclecticism it has come to acquire in current psychiatry.

In summary, critical psychiatry should not be seen as an embarrassing hangover from the 1970s. It can be understood as a non-eclectic, biopsychosocial, neo-Meyerian approach to psychiatry based on Kant's critical philosophy. This position should not be overly polarised in an argument against the biomedical model and recognises that other models, such as the psychodynamic and psychoanalytic, also emphasise psychic aspects. An integrated mind–brain understanding needs to be enriched by a biology that accepts the limitations of a mechanistic interpretation of mental illness and life in general. Critical psychiatry has relevance for modern psychiatry.

## About the author

**Duncan B. Double** is a retired consultant psychiatrist. He blogs on critical psychiatry at www.criticalpsychiatry.blogspot.com.

## References

[1] Double DB. Twenty years of the Critical Psychiatry Network. Br J Psychiatry 2019; 214: 61–2. 30681051 10.1192/bjp.2018.181

[2] Cooper D. Psychiatry and Anti-Psychiatry. Tavistock Publications, 1967.

[3] Roth M. Psychiatry and its critics. Br J Psychiatry 1973; 122: 373–8. 4718272 10.1192/bjp.122.4.373

[4] Tantam D. The anti-psychiatry movement In 150 Years of British Psychiatry: 1841–1991 (eds GE Berrios, H Freeman). Gaskell and Royal College of Psychiatrists, 1991.

[5] Szasz T. Antipsychiatry: Quackery Squared. Syracuse University Press, 2009.

[6] Clare A. Psychiatry in Dissent: Controversial Issues in Thought and Practice. Tavistock Publications, 1976.

[7] Foucault M. History of Madness (ed. J Khalfa; translated by J Murphy, J Khalfa). Routledge, 2006.

[8] Verwey G. Psychiatry in an Anthropological and Biomedical Context. Springer, 1984.

[9] von Feuchtersleben E. The Principles of Medical Psychology (ed. BG Babington; translated by H Evans-Lloyd). Sydenham Society, 1847.

[10] Lesky E. The Vienna Medical School of the 19th Century (Translated from the German). Johns Hopkins University Press, 1976.

[11] Lamb S. Pathologist of the Mind: Adolf Meyer and the Origins of American Psychiatry. Johns Hopkins University Press, 2015.

[12] Double DB. Adolf Meyer's psychobiology and the challenge for biomedicine. Philos Psychiatry Psychol 2007; 14: 331–9.

[13] Engel GL. The need for a new medical model: a challenge for biomedicine. Science 1977; 196: 129–36. 847460 10.1126/science.847460

[14] Ghaemi N. The Rise and Fall of the Biopsychosocial Model: Reconciling Art and Science in Psychiatry. Johns Hopkins University Press, 2010.

[15] Double DB. Eclecticism and Adolf Meyer's functional understanding of mental illness. Philos Psychiatry Psychol 2007; 14: 356–8.

